# Is Model Fitting Necessary for Model-Based fMRI?

**DOI:** 10.1371/journal.pcbi.1004237

**Published:** 2015-06-18

**Authors:** Robert C. Wilson, Yael Niv

**Affiliations:** 1 Department of Psychology and Cognitive Science Program, University of Arizona, Tucson Arizona, United States of America,; 2 Princeton Neuroscience Institute, Princeton University, Princeton, New Jersey, United States of America; University of Oxford, UNITED KINGDOM

## Abstract

Model-based analysis of fMRI data is an important tool for investigating the computational role of different brain regions. With this method, theoretical models of behavior can be leveraged to find the brain structures underlying variables from specific algorithms, such as prediction errors in reinforcement learning. One potential weakness with this approach is that models often have free parameters and thus the results of the analysis may depend on how these free parameters are set. In this work we asked whether this hypothetical weakness is a problem in practice. We first developed general closed-form expressions for the relationship between results of fMRI analyses using different regressors, e.g., one corresponding to the true process underlying the measured data and one a model-derived approximation of the true generative regressor. Then, as a specific test case, we examined the sensitivity of model-based fMRI to the learning rate parameter in reinforcement learning, both in theory and in two previously-published datasets. We found that even gross errors in the learning rate lead to only minute changes in the neural results. Our findings thus suggest that precise model fitting is not always necessary for model-based fMRI. They also highlight the difficulty in using fMRI data for arbitrating between different models or model parameters. While these specific results pertain only to the effect of learning rate in simple reinforcement learning models, we provide a template for testing for effects of different parameters in other models.

## Author Summary

In recent years, model-based fMRI has emerged as a powerful technique in psychology and neuroscience. With this method, computational models of behavior can be leveraged to identify where, whether and how different algorithms are implemented in the brain. Yet this approach seems to have an Achilles heel in that the models frequently have free parameters, and errors in setting these parameters could lead to errors in interpretation of the data. Here we asked whether this potential weakness, in theory, is an actual weakness in practice. In particular, we tested whether errors in estimating participants’ learning rate in a trial-and-error reinforcement learning setting would have adverse effects on identifying the neural substrates of the learning process. Amazingly, it turns out that even gross errors in the learning rate lead to only minute changes in the neural results. The good news is that precise identification of free parameters is not always necessary; the corollary bad news is that it may be harder to identify the precise computational roles of different brain areas than we had previously appreciated. Based on our analytical results, we offer suggestions for designing experiments that maximize or minimize sensitivity to model parameters, as needed.

## Introduction

The advent of fMRI revolutionized psychology as it allowed, for the first time, the noninvasive mapping of human cognition. Despite this progress, traditional fMRI analyses are limited in that they can, for the most part, only ascertain the involvement of an area in a task but not its precise *role* in that task. Recently, model-based fMRI methods have been developed to overcome this limitation by using computational models of behavior to shed light on latent variables of the models (such as prediction errors) and their mapping to neural structures. This approach has led to important insights into the algorithms employed by the brain and has been particularly successful in understanding the neural basis of reinforcement learning (e.g. [1–13]).

In a typical model-based fMRI analysis, one first specifies a model that describes the hypothesized cognitive processes underlying the behavior in question. Typically these models have one or more free parameters (e.g. learning rate in a model of trial-and-error learning). These parameters must be set to fully specify the model, which is commonly done by fitting them to the observed behavior [14]. For instance, given the model, one can find subject-specific learning rates that best explain the subject’s behavioral choices. The fully specified model is then used to generate trial-by-trial measures of latent variables in the model (e.g. action values and prediction errors) that can be regressed against neural data in order to find areas whose activity correlates with these variables in the brain.

One potential weakness of this approach is the requirement for model fitting. In many cases, the data are insufficient to precisely identify the parameter values. This can be due to limited number of trials, interactions between parameters that make them hard to disentangle [14] or lack of behavior that can be used for the fitting process (e.g., in some Pavlovian conditioning experiments). Thus a key question is: How important is the model fitting step? In other words, to what extent is model-based fMRI sensitive to errors in parameter estimation? The answer to this question will determine how hard we should work to obtain the best possible parameter fits, and will affect not only how we analyze data, but also how we design experiments in the first place.

Here we show how this question can be addressed, by analyzing the sensitivity of model-based fMRI to the learning rate parameter in simple reinforcement learning tasks. We provide analytical bounds on the sensitivity of the model-based analysis to errors in estimating the learning rate, and show through simulation how value and prediction error signals generated with one learning rate would be interpreted by a model-based analysis that used the wrong learning rate. Amazingly, we find that the results of model-based fMRI are remarkably robust to settings of the learning rate to the extent that, in some situations, setting the parameters of the model as far as possible from their true value barely affects the results. This theoretical prediction of robustness is borne out by analysis of fMRI data from two recent experiments.

Our findings are both good and bad news for model-based fMRI. The good news is that it is robust, thus errors in the learning rate will not dramatically change the results of studies seeking to localize a particular signal. The bad news, however, is that model-based fMRI is insensitive to differences in parameters, which means that one should use extreme caution when attempting to determine the computational role of a neural area (e.g., when asking whether a brain area corresponds to an outcome signal or a prediction error signal). In the Discussion we consider the extent to which this result generalizes to other parameters and other models and offer suggestions to diagnose parameter sensitivity in other models.

## Methods

### Ethics statement

Both experiments were approved by their respective institutions. The experiment in [10] was approved by the Institutional Review Board of the California Institute of Technology. The experiment in [3] was approved by Ethics Committee at University College London. In both cases participants gave informed consent in writing.

### Theoretical analysis

We begin by laying out a formal analysis of the sensitivity of model-based fMRI to model parameters. The rationale behind the mathematical derivations below is as follows. Assume that there is some signal in the brain (corresponding to some ‘ground truth’ regressor **x**_*g*_) that we have a noisy measurement of (e.g., via fMRI). We first derive the somewhat intuitive result that if we analyze the brain data with a different, incorrect regressor **x**_*f*_ (where the subscript, *f*, denotes that the regressor is derived from our model with *fit* parameter values), the quality of our results depends on the correlation between the ground truth regressor and the incorrect regressor, *ρ*(**x**_*g*_, **x**_*f*_).

To assess the sensitivity of model-based fMRI to errors in parameter estimation, we then focus on trial-and-error learning tasks. We assume a ground truth regressor derived from a reinforcement learning model with the learning rate parameter set to its true (though unknown) value, and analyze the correlation between this regressor and one that is derived from the same model but with a different setting of the learning rate, for some of the most commonly used task designs. Finally, we illustrate and flesh out the implications of these analytical results using both simulated and empirical data in the Results.

#### The effect of an incorrect regressor on fMRI analysis

Assume a ‘ground truth’ regressor **x**_*g*_ = (*x*_*g*1_, *x*_*g*2_, …, *x*_*gT*_) (where *x*_*gt*_ is the size of the variable of interest at time point *t*) that underlies the activity in a brain region, such that the measured signal in this region takes the form

$$\begin{array}{c} Y=\beta x_{g}+\epsilon , \end{array}$$ (1)

with *β* being a coefficient that controls the size of the effect and *ϵ* being zero-mean noise. What would be the magnitude of the estimated regression coefficient $\hat{\beta }$ if we analyzed the brain data using an incorrect regressor, **x**_*f*_ (for example one that is derived from an incorrect model, or from the correct model with the wrong setting of the free parameters)? Using ordinary least squares regression, we have

$$\begin{array}{ccc} \hat{\beta } & = & {\left(x_{f}^{T}x_{f}\right)}^{-1}x_{f}^{T}Y\\ & = & {\left(x_{f}^{T}x_{f}\right)}^{-1}\left(x_{f}^{T}\beta x_{g}\right)\\ & = & \beta \rho \left(x_{g},x_{f}\right)\frac{\sigma (x_{g})}{\sigma (x_{f})} \end{array}$$ (2)

where $\sigma (\text{x})=\sqrt{\frac{{\text{x}}^{T}\text{x}}{T}}$ is the standard deviation of regressor **x**, $\rho ({\text{x}}_{g},{\text{x}}_{f})=\frac{{\text{x}}_{g}^{T}{\text{x}}_{f}}{T\sigma ({\text{x}}_{g})\sigma ({\text{x}}_{f})}$ is the correlation coefficient between **x**_*g*_ and **x**_*f*_, and *T* is the number of data points in the regression. Thus, if we normalize the regressors to have unit variance, i.e. *σ*(**x**_*g*_) = *σ*(**x**_*f*_) = 1, then $\hat{\beta }$ is related to the ground truth regression coefficient through the correlation between the two regressors:

$$\begin{array}{c} \hat{\beta }=\beta \rho \left(x_{g},x_{f}\right) \end{array}$$ (3)

Thus, the more correlated the fit regressor is to the true regressor, the larger the regression coefficient for the fit regressor, $\hat{\beta }$. How does this affect the statistical significance of $\hat{\beta }$, that is, the results of a statistical analysis that asks whether $\hat{\beta }$ is reliably different from zero? To answer this question, we must compute $\hat{t}$, the Student *t* statistic of $\hat{\beta }$ relative to the null hypothesis $\hat{\beta }=0$. Making the further simplifying assumption that the fMRI noise, *ϵ*, is Gaussian with variance $\sigma_{fMRI}^{2}$, we have

$$\begin{array}{c} \hat{t}=\frac{\hat{\beta }}{s(\hat{\beta })} \end{array}$$ (4)

where $s(\hat{\beta })$ is standard error of $\hat{\beta }$. For simple regression, $s(\hat{\beta })$ can be written in terms of the standard deviation of the regression residuals, $\hat{\epsilon }$, as

$$\begin{array}{c} s\left(\hat{\beta }\right)=\frac{1}{\sqrt{T-2}}\frac{\sigma (\hat{\epsilon })}{\sigma (x_{f})}. \end{array}$$ (5)

To compute the standard deviation of the residuals we first note that, by definition,

$$\begin{array}{ccc} \hat{\epsilon } & = & Y-\hat{\beta }x_{f}\\ & = & \beta x_{g}+\epsilon -\beta \rho \left(x_{g},x_{f}\right)x_{f}. \end{array}$$ (6)

Thus, because the residuals have zero mean

σ(ϵ^)2=ϵ^Tϵ^T=1T(β2xgTxg+ϵTϵ+β2ρ(xg,xf)2xfTxf−2β2ρ(xg,xf)xfTxg+2βxgTϵ−2βρ(xg,xf)xfTϵ)=σfMRI2+β2(1-ρ(xg,xf)2) (7)

where we have used the fact that ${\text{x}}_{g}^{T}\epsilon \approx 0$ and ${\text{x}}_{f}^{T}\epsilon \approx 0$ to cancel out the terms in the second line, and the definition of the correlation coefficient to make the simplification in the third line. Combining this expression with Eqs 4 and 5 and keeping in mind that *σ*(**x**) = 1 allows us to write down the *t* statistic as

$$\begin{array}{ccc} \hat{t} & = & \beta \rho \left(x_{g},x_{f}\right)\sqrt{\frac{T-2}{\sigma_{fMRI}^{2}+\beta^{2}\left(1-\rho {\left(x_{g},x_{f}\right)}^{2}\right)}}\\ & = & \rho \left(x_{g},x_{f}\right)\text{CNR}\sqrt{\frac{T-2}{1+{\text{CNR}}^{2}\left(1-\rho {\left(x_{g},x_{f}\right)}^{2}\right)}} \end{array}$$ (8)

where CNR = *β*/*σ*_*fMRI*_ denotes the ‘contrast-to-noise’ ratio [15]—the ratio between the strength of the fMRI signal, *β*, and the standard deviation of the fMRI noise, *σ*_*fMRI*_. Note that this *t* statistic, like the regression coefficient, $\hat{\beta }$, is a function of the correlation between the ground truth and incorrect regressors, *ρ*(**x**_*g*_, **x**_*f*_), as well as the contrast-to-noise ratio, CNR, and the number of data points in the regression, *T*.

#### Correlations between regressors for different parameter settings in a Pavlovian task

We now turn to analyzing the effects of incorrectly specifying free parameters on model-based fMRI. For this, we concentrate on the particular case of reinforcement learning models. These models, which have formed the bulk of model-based fMRI, attempt to predict how values of different options, as computed by the subject, change through experience. One important free parameter in such learning scenarios is the *learning rate* by which values are updated as a result of prediction errors [16]. We assume that there is some ground truth setting of this parameter that corresponds to the true learning rate of the subject, however, this setting is unknown and our fMRI analysis might utilize an incorrect learning rate. Thus to assess the quality of the results we may hope to obtain from the fMRI analysis, we analyze the properties of *ρ*(**x**_*g*_, **x**_*f*_) assuming two regressors that are generated from the same model using different learning rates.

For simplicity, we concentrate on Pavlovian tasks in which subjects experience the rewards associated with different options passively, and use the Rescorla-Wagner learning rule [17]. On each trial, *t*, subjects are presented with a reward *r*_*t*_ and learn to estimate a value *V*_*t*+1_ that represents the reward predicted on the next trial. These values are updated on every trial according to

$$\begin{array}{c} V_{t+1}=V_{t}+\alpha \delta_{t} \end{array}$$ (9)

where *α* is the learning rate and

$$\begin{array}{c} \delta_{t}=r_{t}-V_{t} \end{array}$$ (10)

is the prediction error quantifying the difference between the actual and predicted reward on the current trial. We denote the ground truth learning rate as *α*_*g*_, and the fit learning rate as *α*_*f*_. Accordingly, we denote the vector of values learned with learning rate *α*_*i*_ as **V**_*i*_ = (*V*_*i*1_, *V*_*i*2_, …, *V*_*iT*_) and the corresponding vector of prediction errors as **δ**_*i*_.

Model-based fMRI studies of reinforcement learning typically use values and prediction errors as regressors for neural activity [18]. Thus, in order to determine the sensitivity of the results to the accuracy of the parameter fits, we need to compute the correlation coefficients *ρ*(**V**_*g*_, **V**_*f*_) and *ρ*(**δ**_*g*_, **δ**_*f*_). To do this, we first note that in the general case, the values and prediction errors computed according to Eqs 9 and 10 will not have zero mean or unit variance. This does not invalidate the previously derived results but does require us to work with the more general form of the correlation coefficient

$$\begin{array}{c} \rho \left(a,b\right)=\frac{\text{cov}(a,b)}{\sigma (a)\sigma (b)} \end{array}$$ (11)

where cov(**a**, **b**) is the covariance between vectors **a** and **b** defined by

$$\begin{array}{c} \text{cov}\left(a,b\right)=\frac{a^{T}b}{T}-\mu \left(a\right)\mu \left(b\right). \end{array}$$ (12)

Here *μ*(**a**) is the mean of vector **a**, and *σ*(**a**), the standard deviation of an uncentered regressor, is given by

$$\begin{array}{c} \sigma \left(a\right)=\sqrt{\frac{a^{T}a}{T}-\mu {\left(a\right)}^{2}}. \end{array}$$ (13)

Thus to compute the correlation coefficients *ρ*(**V**_*g*_, **V**_*f*_) and *ρ*(**δ**_*g*_, **δ**_*f*_) we must compute the mean, variance and covariance of the value and prediction error regressors. These statistics turn out to be completely determined by the learning rates and the statistics of the reward vector, **r**.

In particular, for the value regressors it can be shown that

$$\begin{array}{ccc} \mu (V_{i}) & \approx & \mu (r)\\ \sigma {\left(V_{i}\right)}^{2} & \approx & \frac{\alpha_{i}}{2-\alpha_{i}}(\mu \left(r^{2}\right)+\frac{2}{T}\sum_{\Delta =1}^{T-1}\left(T-\Delta \right){\left(1-\alpha_{i}\right)}^{\Delta }R_{\Delta }\left(r\right))-\mu {\left(r\right)}^{2}\\ \text{cov}(V_{i},V_{j}) & \approx & \frac{\alpha_{i}\alpha_{j}}{\alpha_{i}+\alpha_{j}-\alpha_{i}\alpha_{j}}(\mu \left(r^{2}\right)+\frac{1}{T}\sum_{\Delta =1}^{T-1}({\left(1-\alpha_{i}\right)}^{\Delta }+{\left(1-\alpha_{j}\right)}^{\Delta })\left(T-\Delta \right)R_{\Delta }\left(r\right))-\mu {\left(r\right)}^{2} \end{array}$$ (14)

where the approximations hold in the limit of many trials and *R*_Δ_(**r**) is the (uncentered) autocorrelation of **r** at delay Δ defined as

$$\begin{array}{c} R_{\Delta }\left(r\right)=\frac{1}{T-\Delta }\sum_{a=1}^{T-\Delta }r_{a}r_{a+\Delta }. \end{array}$$ (15)

Equivalently, for the prediction errors we have

$$\begin{array}{ccc} \mu (\delta_{i}) & \approx & 0\\ \sigma {\left(\delta_{i}\right)}^{2} & \approx & \frac{2}{2-\alpha_{i}}(\mu \left(r^{2}\right)-\sum_{\Delta =1}^{T-1}\alpha_{i}{\left(1-\alpha_{i}\right)}^{\Delta -1}(1-\frac{\Delta }{T})R_{\Delta }\left(r\right))\\ \text{cov}(\delta_{i},\delta_{j}) & \approx & \frac{1}{\alpha_{i}+\alpha_{j}-\alpha_{i}\alpha_{j}}(\left(\alpha_{i}+\alpha_{j}\right)\mu \left(r^{2}\right)-\sum_{\Delta =1}^{T-1}(\alpha_{i}^{2}{\left(1-\alpha_{i}\right)}^{\Delta -1}+\alpha_{j}^{2}{\left(1-\alpha_{j}\right)}^{\Delta -1})(1-\frac{\Delta }{T})R_{\Delta }\left(r\right)) \end{array}$$ (16)

See the Supplementary Information for detailed derivation of these equations. It is important to note that the approximations in Eqs 14 and 16 only hold when the number of trials, *T*, is large relative to the reciprocal of the run lengths (1/*α*_*g*_ and 1/*α*_*f*_). This approximation simplifies the expressions greatly, by removing the dependence on initial values, but we urge caution in interpreting the results when both *T* and the learning rates are small. In particular, this implies that that our analysis holds for non-zero learning rates only.

Eqs 14 and 16 imply that to compute the required correlations we only need the statistics of the rewards: *μ*(**r**), *μ*(**r** ^2^) and *R*_Δ_(**r**). The exact form of these averages depends on the dynamics of the reward-generating process in the experiment. In the Results section we consider two commonly used experimental designs.

### fMRI experiments

To test our theoretical predictions we used data from two different experiments corresponding to two different reward dynamics: fixed and drifting. In the following sections we briefly describe the two experiments along with details of our analyses. More precise descriptions of each experiment can be found in the original papers, also available as part of the supplementary information (S1 Dataset).

#### Fixed reward distribution

We used data from Niv, Edlund, Dayan & O’Doherty [10]. Preprocessing of the fMRI data were performed by the original authors, as described in [10]. In this experiment, 16 subjects made a series of choices between stimuli that paid out different amounts of monetary reward. There were five possible stimuli: two options paid out 0¢ with 100% probability, one paid out 20¢, one paid out 40¢, and one paid out either 0¢ or 40¢ with 50% probability (henceforth the risky 0/40 stimulus). The experiment involved two types of trials, intermingled: on ‘choice trials’ subjects were required to choose between two stimuli, while on ‘forced trials’ subjects were presented with only one of the five stimuli and had to choose it. These forced trials ensured that subjects continued to experience all of the stimuli regardless of their subjective value (for example, the 0¢ stimuli were very rarely chosen on choice trials). Choices were made immediately after the stimuli appeared on screen and reward feedback was given 5s after the choice.

Reinforcement learning theory predicts that there would be two prediction errors on each trial: one at the time of stimulus onset/choice, equal to the value of the to-be-chosen stimulus **V**, and one at the time of reward, given by the difference between reward outcome and the value of the chosen option, **r** − **V**. Because subjects were trained on the task prior to the scan, we assumed that they knew the values for the constantly rewarding stimuli, and therefore concentrated on learning estimated values for the 0/40 stimulus, for which rewards were probabilistic. Note that the simple reinforcement-learning model we use is slightly different from the best-fitting model used in [10], which involved two different learning rates for positive and negative prediction errors. We used the simpler model to maintain consistency with our theoretical analysis. However, in any case our results show that such a difference in the model would not affect the regressors and the neural results to a large extent.

We focused our analysis on fMRI activations in the nucleus accumbens (NAc), an area whose BOLD activity has been repeatedly shown to correlate with prediction errors for both primary [2, 18–24] and monetary rewards [3, 10, 25–29], putatively due to the strong dopaminergic afferents to that area. We used average BOLD signals extracted by the original authors from anatomically defined regions of interest (ROIs) in the left and right NAc, and regressed this signal vector, **Y**, against parametric regressors for value and prediction error of the risky 0/40 stimulus as well as regressors for variables of less interest such as event onsets, value of the certain options and nuisance variables such as head motion and scanner drift. In keeping with our theoretical analysis, we mean-centered all model-based regressors and normalized them to have a standard deviation of 1. We repeated this analysis using different settings of the learning rate between 0.01 and 1, in steps of 0.01.

#### Drifting reward distribution

For the drifting reward distribution, we analyzed data from Daw, O’Doherty, Dayan, Seymour & Dolan [3]. Preprocessing of the fMRI data was done by the original authors, as described in [3]. In this experiment, 16 subjects performed two blocks of 150 choices between four options. Each option paid out probabilistic rewards sampled from a Gaussian distribution with a drifting mean and a noise standard deviation *σ*_*n*_ = 4. The drifting mean *m*_*t*_ on trial *t* was updated according to

$$\begin{array}{c} m_{t+1}=\gamma m_{t}+50\left(1-\gamma \right)+n_{t} \end{array}$$ (17)

where *n*_*t*_ was Gaussian random noise with drift standard deviation, *σ*_*d*_ = 2.8 and *γ* = 0.9836 was the decay rate that ensured that the mean decayed towards 50.

As in the experiment with fixed reward distributions [10], presentation of the stimulus and outcome were separated in time, and as before, we expected the signal at the stimulus to reflect the value of the to-be-chosen stimulus, **V**, and the signal at the outcome to reflect the prediction error **r** − **V**. We thus used a GLM with 5 regressors: two stick regressors, one at the onset of stimuli and one at the onset of the outcomes, a **V** parametric modulation on stimulus onset, a prediction error parametric modulation on outcome onset, and a parametric modulation on outcome onset by the magnitude of outcome itself, **r**. Because we were primarily interested in the value signal, we focused on data from an ROI in the ventromedial prefrontal cortex (vmPFC). Specifically, we used an ROI centered at (-3, 33, -6), a location that was reported in [3] to correlate strongly with choice probability, which is closely related to chosen value. As before, we analyzed this ROI with GLMs created using a variety of different learning rates between 0.01 and 1, in steps of 0.01.

## Results

In the Methods, we developed general expressions that describe how changes in the learning rate affect model-based fMRI in simple reinforcement learning tasks. In particular, we showed that regression coefficients when using a model-based regressor, and their corresponding Student *t* values, depend on three factors: the contrast-to-noise ratio (CNR) in the signal, the number of data points in the regression, and *ρ*(**x**_*g*_, **x**_*f*_)—the correlation between the regressor generated with the fit parameter values, **x**_*f*_, and a regressor generated with the ground truth parameter values, **x**_*g*_.

We now investigate how these factors play out in two cases with qualitatively different reward dynamics: a reward distribution that is fixed throughout the experiment, or one that changes over time. In both cases we show that model-based fMRI analysis of value and prediction error signals is relatively insensitive to the setting of the learning rate parameter and that this insensitivity can be, to a certain extent, manipulated by altering the design of the task.

### Fixed reward distribution

**Fig 1:**
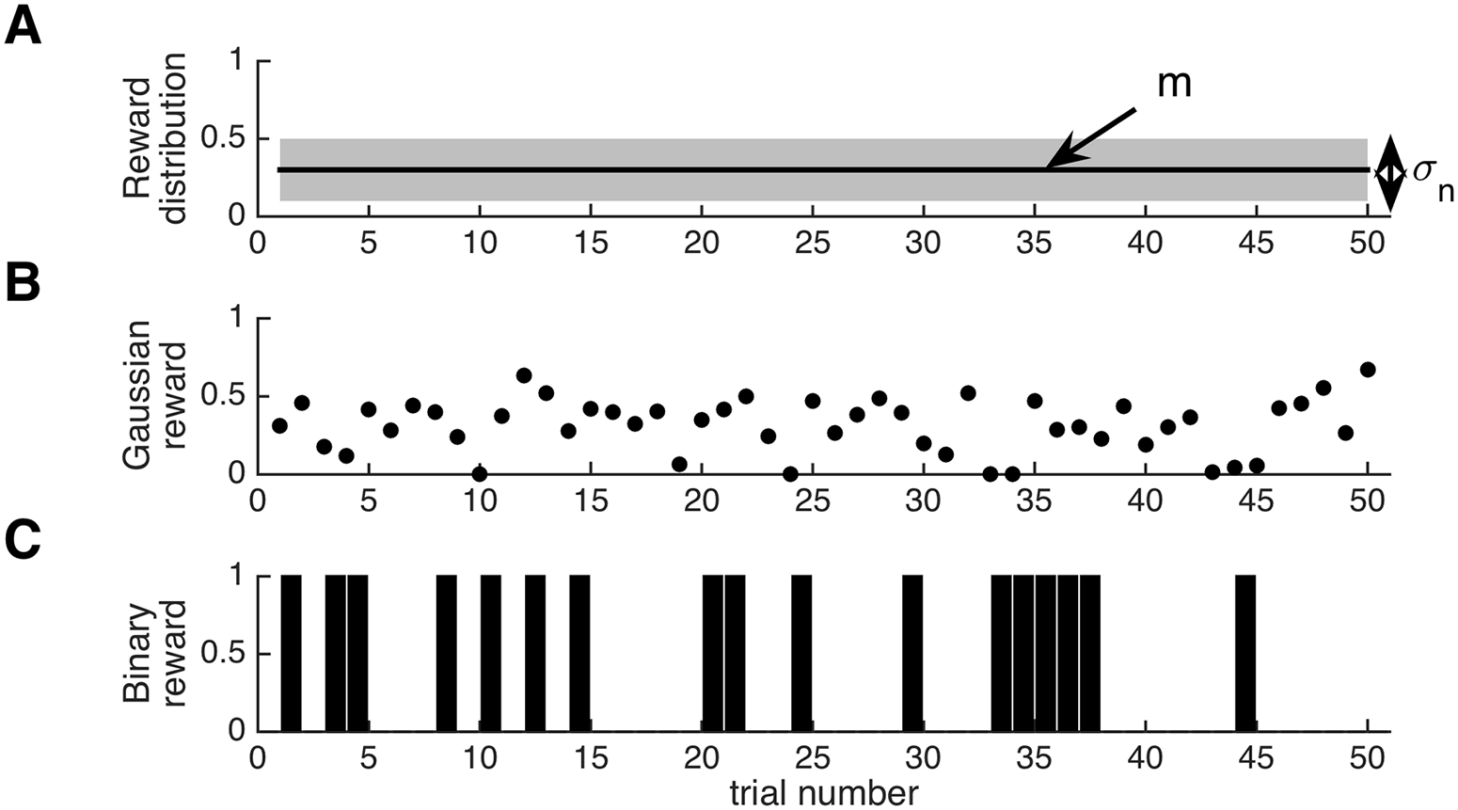
Illustration of a fixed reward distribution. (A) Schematic of a general reward distribution that is fixed over time, showing the relevant parameters: the mean *m* and standard deviation *σ*_*n*_. (B) Example of continuous rewards sampled from a Gaussian distribution with mean *m* = 0.3 and standard deviation *σ*_*n*_ = 0.2. (C) Bar plot showing an example of binary reward data sampled from a Bernoulli distribution with the same mean *m* = 0.3 and standard deviation $\sigma_{n}=\sqrt{m(1-m)}\approx 0.46$.

We first consider a situation in which the reward distribution is fixed throughout the experiment. An example of such a distribution with mean *m* and variance $\sigma_{n}^{2}$ is shown in Fig 1A. In panels B and C of the same figure we show rewards from Gaussian and Bernoulli distributions, but it is important to note that the following theoretical results apply to *any* fixed reward distribution with finite variance.

#### Analytic results

Based on the methods developed above, the sensitivity of a model-based analysis looking for a value signal in the brain depends on the correlation, *ρ*(**V**_*g*_, **V**_*f*_), between the value computed using the ground truth learning rate, **V**_*g*_, and the value computed using the fit learning rate, **V**_*f*_. Likewise prediction error signals depend on *ρ*(**δ**_*g*_, **δ**_*f*_). Moreover, expressions for these correlations rely only on a few statistics of the reward distribution: its mean *μ*(**r**) = *m*, the mean of the squared rewards $\mu ({\text{r}}^{2})=m^{2}+\sigma_{n}^{2}$, and the reward autocorrelation *R*_Δ_(**r**) = *m* ^2^. Given these expressions for the reward statistics, we can compute the sums in Eqs 14 and 16 exactly (as sums of geometric series), leading to the following expressions for the value and prediction error correlations using different learning rates *α*_*g*_ and *α*_*f*_:

$$\begin{array}{ccc} \rho (V_{g},V_{f}) & = & \frac{\sqrt{\alpha_{g}\alpha_{f}\left(2-\alpha_{g}\right)\left(2-\alpha_{f}\right)}}{\alpha_{g}+\alpha_{f}-\alpha_{g}\alpha_{f}}\\ \rho (\delta_{g},\delta_{f}) & = & \frac{\left(\alpha_{g}+\alpha_{f}\right)\sqrt{\left(2-\alpha_{g}\right)\left(2-\alpha_{f}\right)}}{2(\alpha_{g}+\alpha_{f}-\alpha_{g}\alpha_{f})} \end{array}$$ (18)

**Fig 2:**
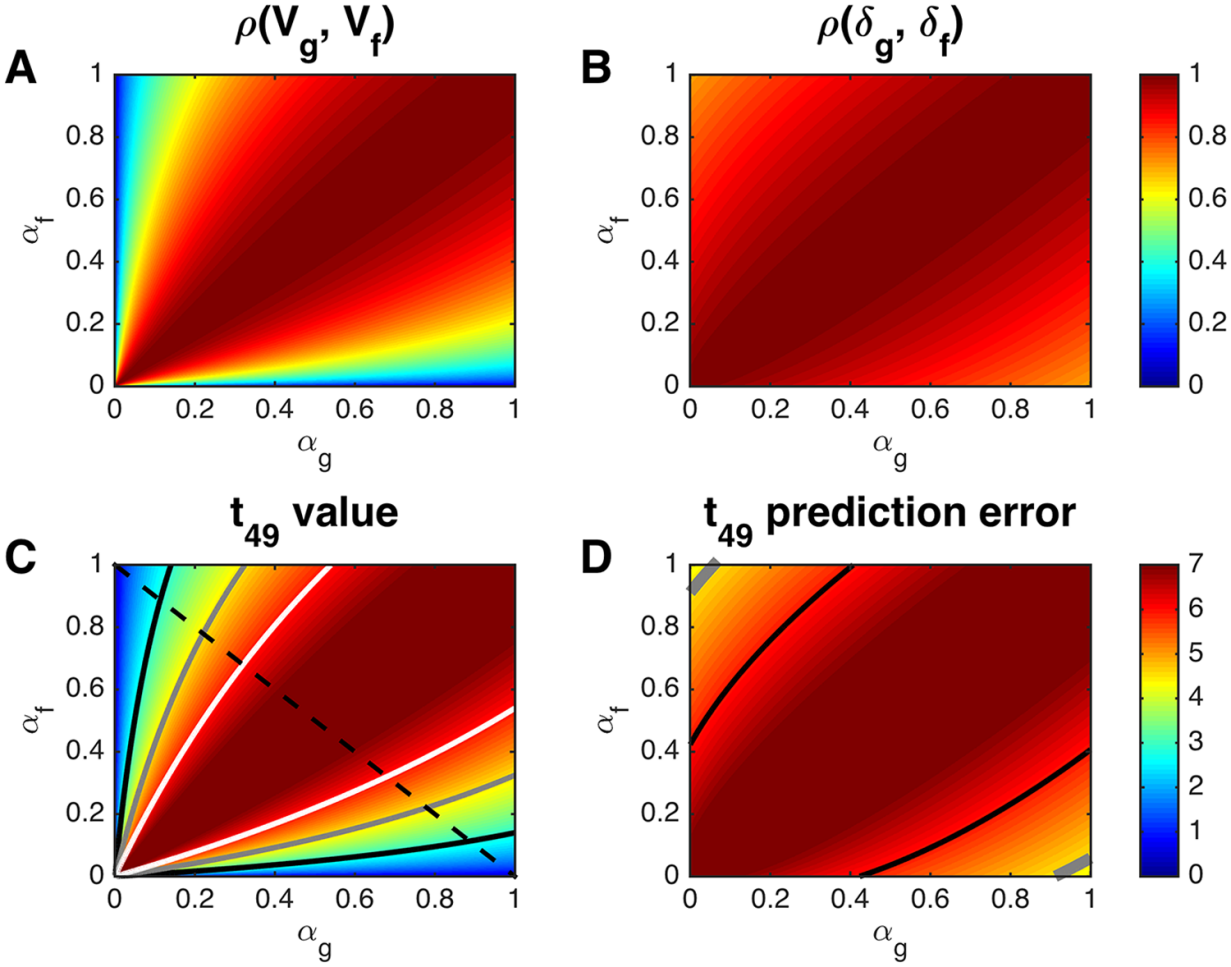
Correlations and *t* statistics for experiments with a fixed reward distribution, computed by evaluating Eq (18) at learning rates between 0.001 and 1, in steps of 0.001. (A) Correlation, *ρ*(**V**_*g*_, **V**_*f*_), between regressors for value as a function of the true, *α*_*g*_, and fitted, *α*_*f*_, learning rates. (B) Correlation between the regressors for prediction error, *ρ*(**δ**_*g*_, **δ**_*f*_). (C,D) Single-subject *t* statistics (assuming 49 degrees of freedom) as a function of the two learning rates for value (C) and prediction error (D). Black, gray and white contours denote significance at *p* = 0.01, *p* = 10^−4^ and *p* = 10^−6^, respectively. Dashed black line in C: values that will be analyzed in more detail in Fig 3.

In Fig 2A and 2B we plot these correlations as functions of the two learning rates. Strikingly, the correlations for both value and prediction error are relatively insensitive to mismatch in learning rates. Indeed, for prediction errors, the minimum possible value of the correlation (at *α*_*g*_ → 0 and *α*_*f*_ → 1 or vice versa) is $1/\sqrt{2}\approx 0.7$. This implies that even in the worst case scenario, when the true learning rate had an extreme value of 0 or 1 and the fit learning rate was as far as possible from the true learning rate, the resulting prediction error regressor would still be highly correlated with the true signal. This result has important implications for the qualitative interpretation of prediction error signals, since with a true learning rate of 0, the ‘prediction error’ is simply the reward signal. Therefore, when the reward distribution is fixed throughout the experiment, prediction errors will always be strongly correlated with the reward signal, making it difficult to tease apart these two neural signals using linear regression.

In Fig 2C and 2D we show the corresponding *t* statistics assuming 49 degrees of freedom (or a very conservative total number of trials, *T* = 50) and a contrast-to-noise ratio, CNR of 1 (a fairly typical value in fMRI experiments [15] and also consistent with the range of CNR values seen in the data sets analyzed here, where CNR was 0.4 in one case and 11 in the other). As can be seen, the dependence of the *t* statistic for the regressor on the true and fit learning rates closely matches that of the regressor correlations. This is because at low CNR the *t* statistic in Eq 8 is approximately proportional to *ρ*(**x**_*g*_, **x**_*f*_). For reward prediction error in particular, all possible values of *α*_*g*_ and *α*_*f*_ result in a significant *t* statistic at *p* < 0.001 (a commonly used uncorrected threshold for significance of prediction error signals in the brain). This result further exemplifies both the strength and the limitation of such an analysis: a neural signal will likely be identified even if model fitting produced especially poor learning rate parameter settings, however, from this regression alone we cannot know for sure that the identified signal is indeed a prediction error signal rather than a reward signal.

**Fig 3:**
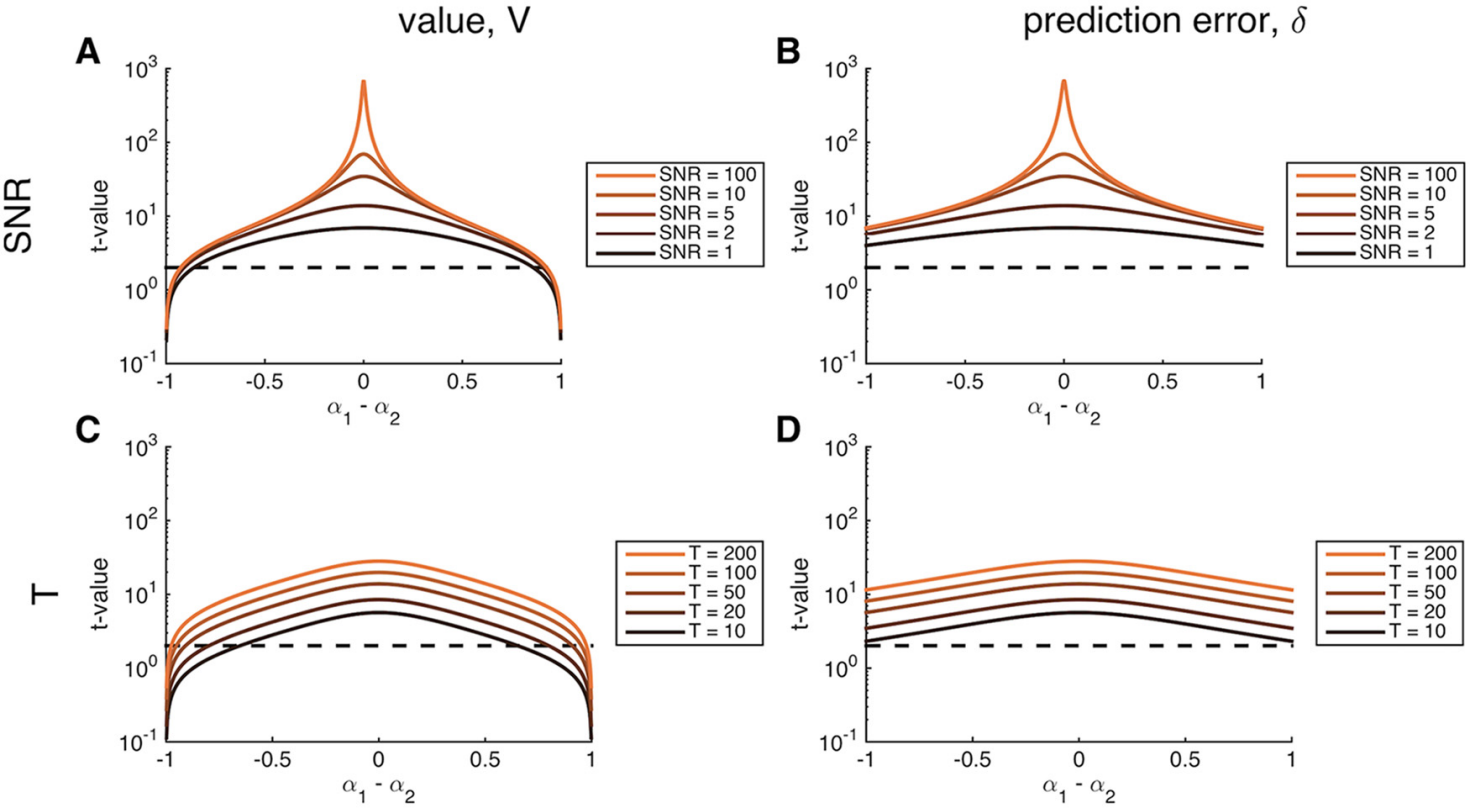
Effect of contrast-to-noise ratio, CNR, (A,B) and number of trials, *T*, (C,D) on the *t* values as a function of the difference between *α*_*g*_ − *α*_*f*_ when *α*_*g*_ + *α*_*f*_ = 1, for experiments with a fixed reward distribution. The range of differences is from -0.999 to +0.999 in steps of 0.001. Dashed line: significance at *p* < 0.05 at the single subject level.

In Fig 3 we investigate the effect of CNR and *T* on the *t* statistic more explicitly. For exposition, we focus on the diagonal *α*_*g*_ + *α*_*f*_ = 1 (i.e. along the dashed line in Fig 2C) and plot the results as a function of the difference in learning rates, *α*_*g*_ − *α*_*f*_, with the constraint that *α*_*g*_ + *α*_*f*_ = 1. Panels A and B show the effect of changing the contrast-to-noise ratio from 1 to 100 at fixed *T* = 50 for the value and prediction error regressors, respectively. In both cases, as the contrast-to-noise ratio increases, the curves become increasingly peaked at 0 (i.e. at *α*_*g*_ = *α*_*f*_) indicating greater sensitivity of the resulting *t* statistics to accuracy of the fit learning rate. This shows that, when the underlying signal is strong, model-based fMRI will be much more sensitive to parameter settings than when the signal is weak. Of course, for a given statistical threshold, a higher CNR also results in a wider range of fit learning rates achieving statistical significance. Thus, not surprisingly, high CNR is always better than low CNR in that it makes the result more robust to model-fitting errors in addition to making it easier to use the fMRI signal to inform the parameter search.

In panels C and D we illustrate the effect of changing the number of trials, *T*, from 10 to 200 assuming a fixed CNR = 1. Again, as *T* increases, the *t* statistic becomes increasingly peaked around *α*_*g*_ = *α*_*f*_ (note the logarithmic scale on the y-axis). These results show that the sensitivity of a model-based analysis can also be increased by increasing the number of trials in the experiment.

#### fMRI data

**Fig 4:**
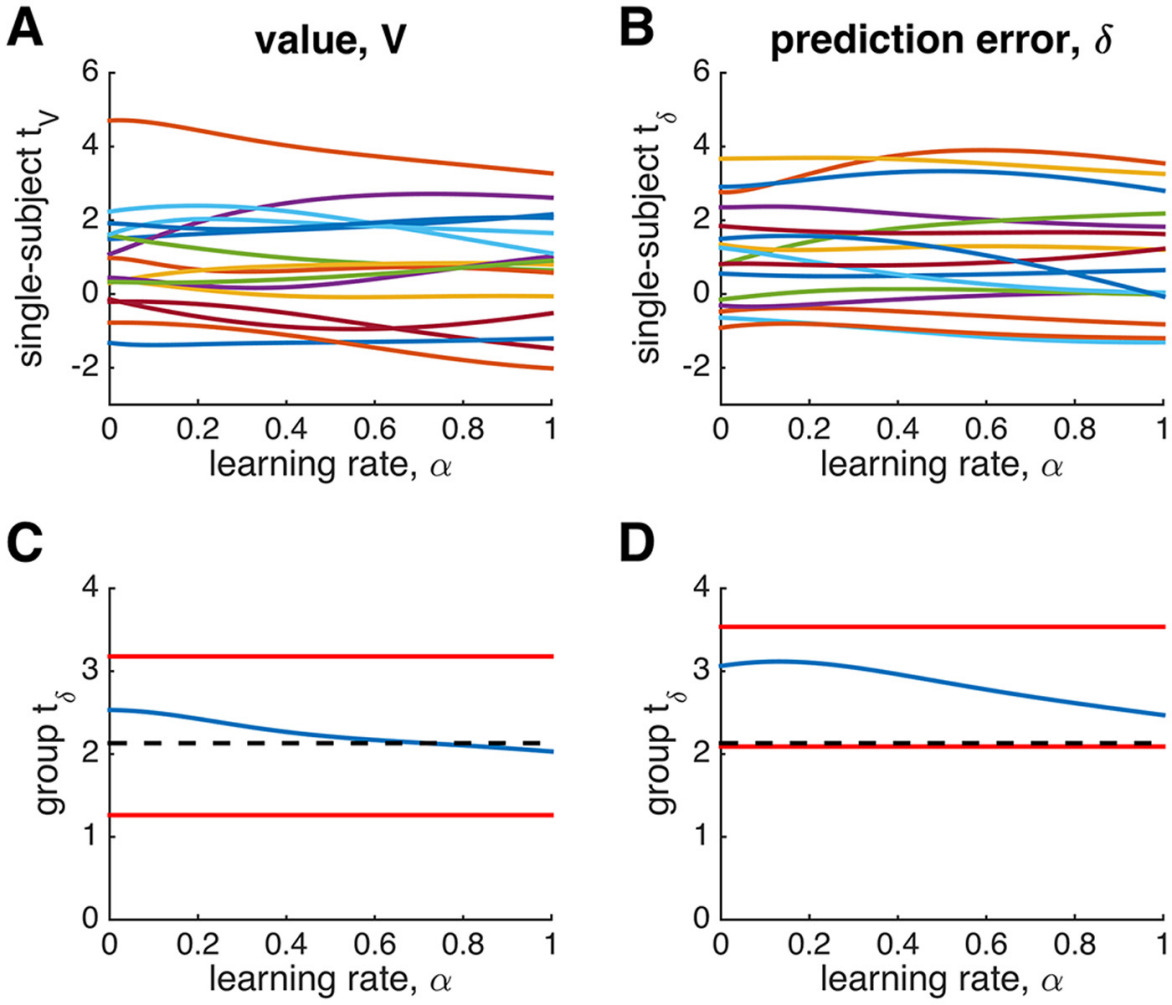
The effect of learning rate on the fMRI signal in the NAc in [10], an experiment with a fixed reward distribution (binary rewards with *p* = 0.5, see Methods). (A) Regression coefficients for value of the chosen option at the time of stimulus onset, as a function of learning rate. Each curve represents a single subject. (B) Single subject regression coefficients for prediction error at the time of reward. Note the relative lack of modulation of the regression coefficient by the value of the learning rate parameter. (C) Group analysis at the time of stimulus showing the group *t* statistic as a function of (group-wise) learning rate (blue). Red lines denote the best and worst case scenarios obtained by taking the value of the learning rate that either maximizes or minimizes the *t* statistic for each subject. Dashed black line: *p* = 0.05 threshold. (D) Group analysis at the time of reward. In all cases, as predicted, the effect of the learning rate parameter is small.

To test these theoretical predictions, we used data from from Niv, Edlund, Dayan & O’Doherty [10]. As Fig 4 shows, consistent with the theory, for both value and prediction error regressors, the regression coefficient depended relatively weakly on the learning rate used to generate the regressor. This was true both for single subjects (Fig 4A and 4B) and at the group level (Fig 4C and 4D).

Although the plots in Fig 4A and 4B are relatively insensitive to parameter value, many of the curves for individual subjects do appear to have a maximum, suggesting that there is a learning rate that best describes the neural data. To evaluate the extent to which these best-fitting learning rates could be estimated, we computed the log likelihood of the fMRI data for a linear model assuming that the data are generated by the a combination of the model-based regressors for value and prediction error plus additive Gaussian noise, for each value of the learning rate parameter. For a linear regression model, the log likelihood has a closed form as follows:

$$\begin{array}{c} LL\left(\alpha \right)=-T(\log(\sqrt{2\pi }\sigma_{r}\left(\alpha \right))+\frac{1}{2}) \end{array}$$ (19)

where *σ*_*r*_(*α*) denotes the standard deviation of the residuals of the linear model with model-based regressors generated using learning rate *α*.

**Fig 5:**
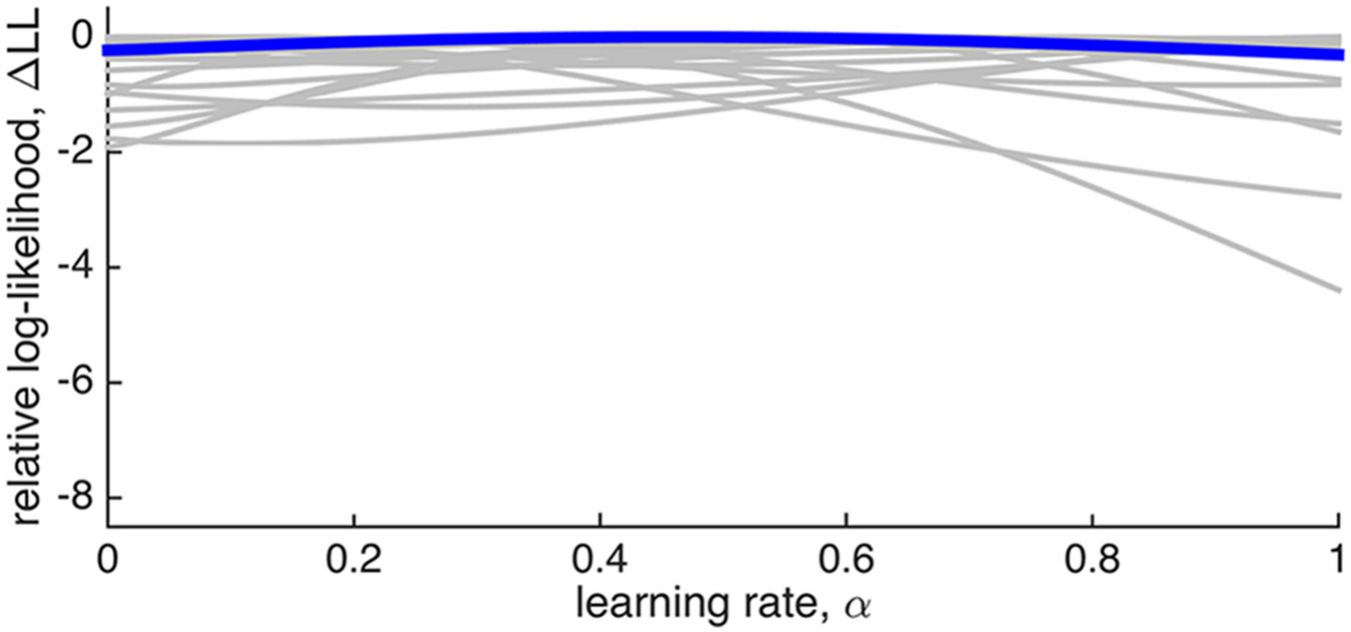
Fit of a linear model comprised of a single model-based regressor generated with different learning rates *α*, to fMRI data in the fixed reward probability experiment [10] as a function of learning rate. Each grey curve corresponds to a different subject and in blue is the mean across subjects. All curves are shifted to have a maximum value of 0. For most subjects the quality of the fit depends only weakly on the learning rate. Note that the y-axis is the same as in Fig 10, to highlight the differences between the sensitivities of the two experiments to the setting of the learning rate parameter.

Fig 5 shows *LL*(*α*) relative to its maximum value, for each subject, Δ*LL*(*α*) = *LL*(*α*) − max_*α*_ *LL*(*α*). This analysis further highlights the insensitivity of these results to learning rate—for most subjects the range of log likelihood between best and worst fits being less than 2, a difference in fit usually considered ‘barely worth mentioning’ [30].

### Drifting reward distribution

**Fig 6:**
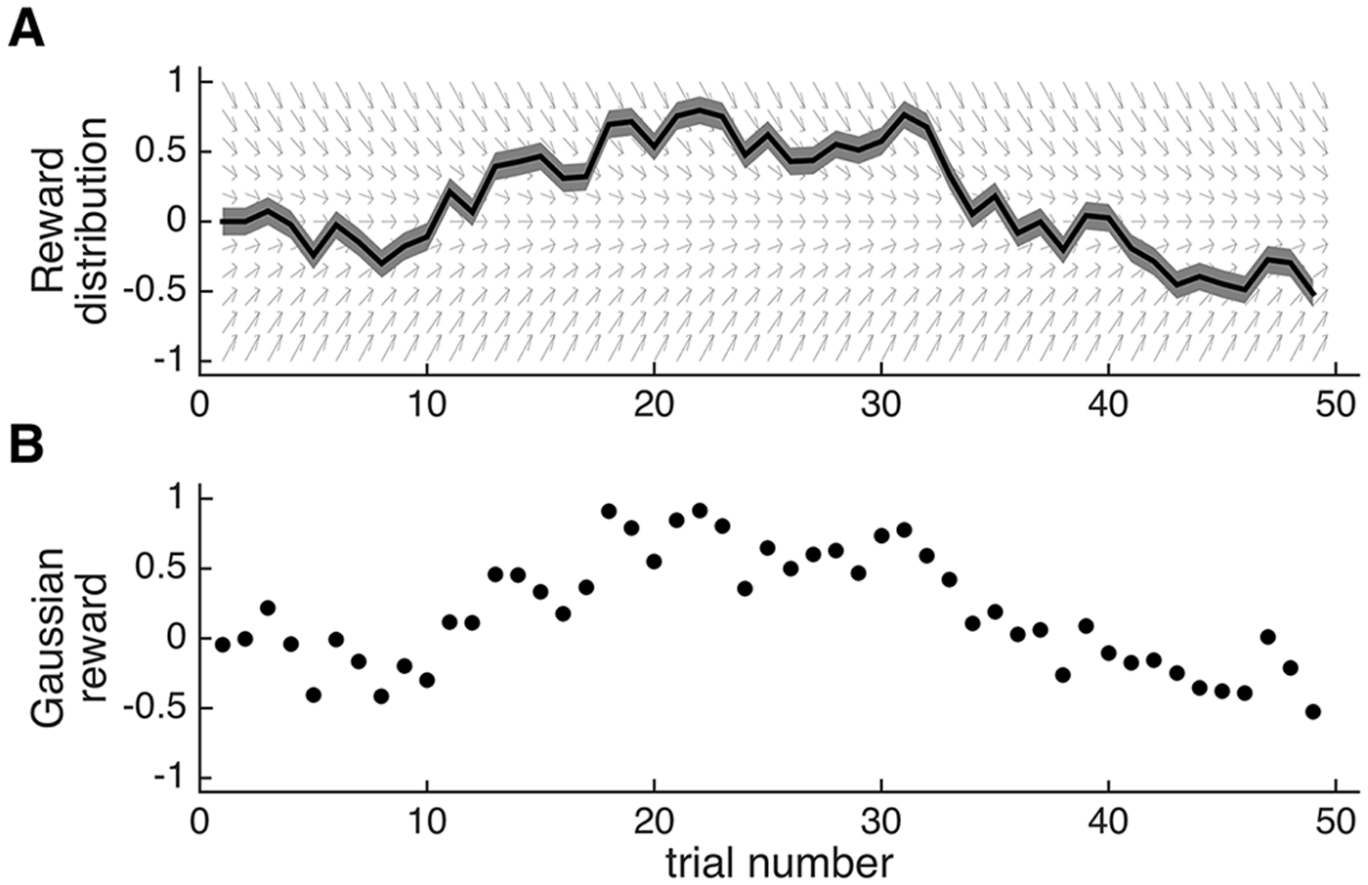
An example drifting reward distribution. (A) Evolution of the mean *m*_*t*_ over time, *t*, diffusing with a drift standard deviation *σ*_*d*_. The decay, *γ*, is indicated by the gray arrows and the shaded region indicates the standard deviation of the Gaussian noise distribution, *σ*_*n*_. (B) A set of rewards sampled from the distribution in panel A.

Our approach can also be applied to scenarios in which the reward distribution is not fixed. To illustrate, we analyze experiments with rewards that are drawn from a Gaussian distribution whose mean, *m*_*t*_, is generated by a discretized Ornstein-Uhlenbeck process (Fig 6) [31]. Specifically, *m*_*t*_, undergoes a random walk defined by

$$\begin{array}{c} m_{t+1}=\gamma m_{t}+n_{t} \end{array}$$ (20)

where *n*_*t*_ is zero mean noise with drift variance $\sigma_{d}^{2}$, and *γ* (< 1) is a decay parameter. Because *γ* is smaller than one, the mean tends to decay to zero over time (illustrated by the arrows in Fig 6A). This helps to keep the means of different options from diverging too far as the experiment progresses.

#### Analytic results

Again, to compute the correlations we require the statistics of the reward distribution. For simplicity, we focus on situations in which *T* is large, where the reward statistics are asymptotically

$$\begin{array}{c} \mu (r)=0 \end{array}$$ (21)

$$\begin{array}{c} \mu \left(r^{2}\right)=\sigma_{n}^{2}+\frac{\sigma_{d}^{2}}{1-\gamma^{2}} \end{array}$$ (22)

$$\begin{array}{c} R_{\Delta }\left(r\right)=\frac{\gamma^{\Delta }\sigma_{d}^{2}}{1-\gamma^{2}}. \end{array}$$ (23)

These reward statistics allow us to compute the sums in Eqs 14 and 16 exactly, leading to the following (slightly messy, but nonetheless fully tractable) expressions for *ρ*(**V**_*g*_, **V**_*f*_) and *ρ*(**δ**_*g*_, **δ**_*f*_):

$$\begin{array}{ccc} \rho (V_{g},V_{f}) & = & \frac{\left(1+(\frac{1}{1-\gamma +\alpha_{g}\gamma }+\frac{1}{1-\gamma +\alpha_{f}\gamma }-1)\frac{\sigma_{d}^{2}}{\left(1-\gamma^{2}\right)\sigma_{n}^{2}}\right)}{\alpha_{g}+\alpha_{f}-\alpha_{g}\alpha_{f}}\sqrt{\frac{\alpha_{g}\alpha_{f}\left(2-\alpha_{g}\right)\left(2-\alpha_{f}\right)}{\left(1+\frac{2}{\left(1-\gamma +\alpha_{g}\gamma \right)}\frac{\sigma_{d}^{2}}{\left(1-\gamma^{2}\right)\sigma_{n}^{2}})(1+\frac{2}{1-\gamma +\alpha_{f}\gamma }\frac{\sigma_{d}^{2}}{\left(1-\gamma^{2}\right)\sigma_{n}^{2}}\right)}}\\ \rho (\delta_{g},\delta_{f}) & = & \frac{\left(\alpha_{g}+\alpha_{f}\right)+(\frac{\alpha_{g}}{1-\gamma +\alpha_{g}\gamma }+\frac{\alpha_{f}}{1-\gamma +\alpha_{f}\gamma })\frac{\sigma_{d}^{2}}{\left(1+\gamma \right)\sigma_{n}^{2}}}{2(\alpha_{g}+\alpha_{f}-\alpha_{g}\alpha_{f})}\sqrt{\frac{\left(2-\alpha_{g}\right)\left(2-\alpha_{f}\right)}{\left(1+\frac{1}{1-\gamma +\alpha_{g}\gamma }\frac{\sigma_{d}^{2}}{\left(1+\gamma \right)\sigma_{n}^{2}})(1+\frac{1}{1-\gamma +\alpha_{f}\gamma }\frac{\sigma_{d}^{2}}{\left(1+\gamma \right)\sigma_{n}^{2}}\right)}}. \end{array}$$ (24)

**Fig 7:**
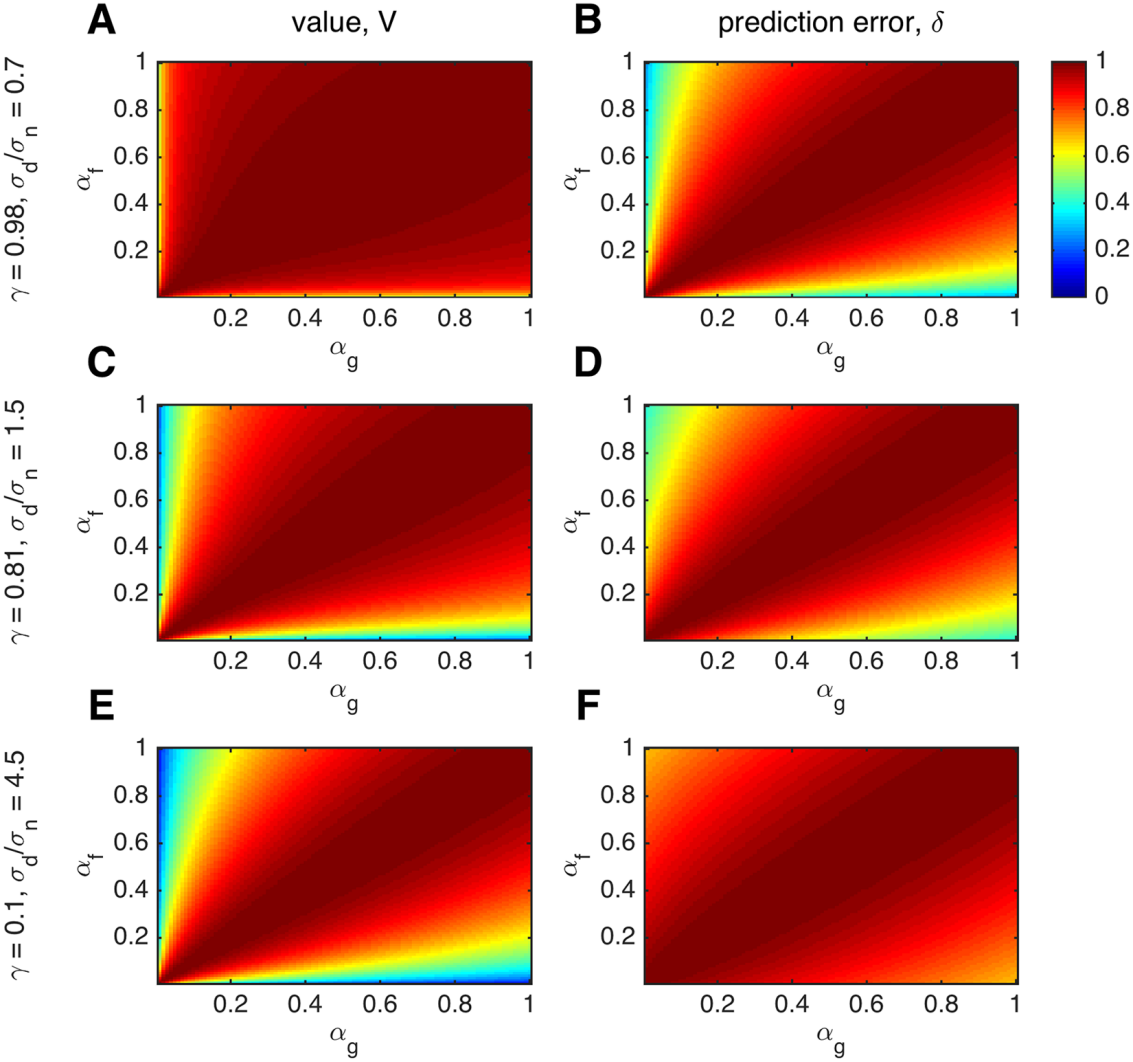
Correlations between model based regressors derived using different learning rates, in an experiment with drifting rewards, for three different settings of the decay of the reward mean to 0, *γ*, and the drift-to-noise ratio of the reward mean, *σ*_*d*_/*σ*_*n*_. Plots were generated by evaluating Eq (24) for learning rates between 0.001 and 1 in steps of 0.001. (A,B) When *γ* is high (0.98) and *σ*_*d*_/*σ*_*n*_ is low (0.7), values are not sensitive to fit learning rate, but prediction errors are sensitive. (C,D) Intermediate *γ* and *σ*_*d*_/*σ*_*n*_ lead to intermediate sensitivity of both value and prediction error to learning rate. (E, F) When *γ* is low (0.1) and *σ*_*d*_/*σ*_*n*_ is high (4.5), the results mimic those obtained with a fixed reward distribution (values are more sensitive to fit learning rate than are prediction errors, compare Fig 2A and 2B).

Note that these are functions of only two parameters of the reward distribution: the decay, *γ*, and the ratio of drift variance to noise variance, $\sigma_{d}^{2}/\sigma_{n}^{2}$. When using a drifting reward probability, these experimentally determined parameters exert much greater control over the form of the correlations than can be achieved when reward probability is fixed. This control is demonstrated in Fig 7 where we tuned these two parameters to achieve sensitivity to prediction errors (panels A,B), value (panels E,F), or both (panels C,D). The parameters in panels A and B, *γ* = 0.98 and *σ*_*d*_/*σ*_*n*_ = 0.7, closely match those in the experiment of Daw et al. [3], whose neural results we discuss below.

**Fig 8:**
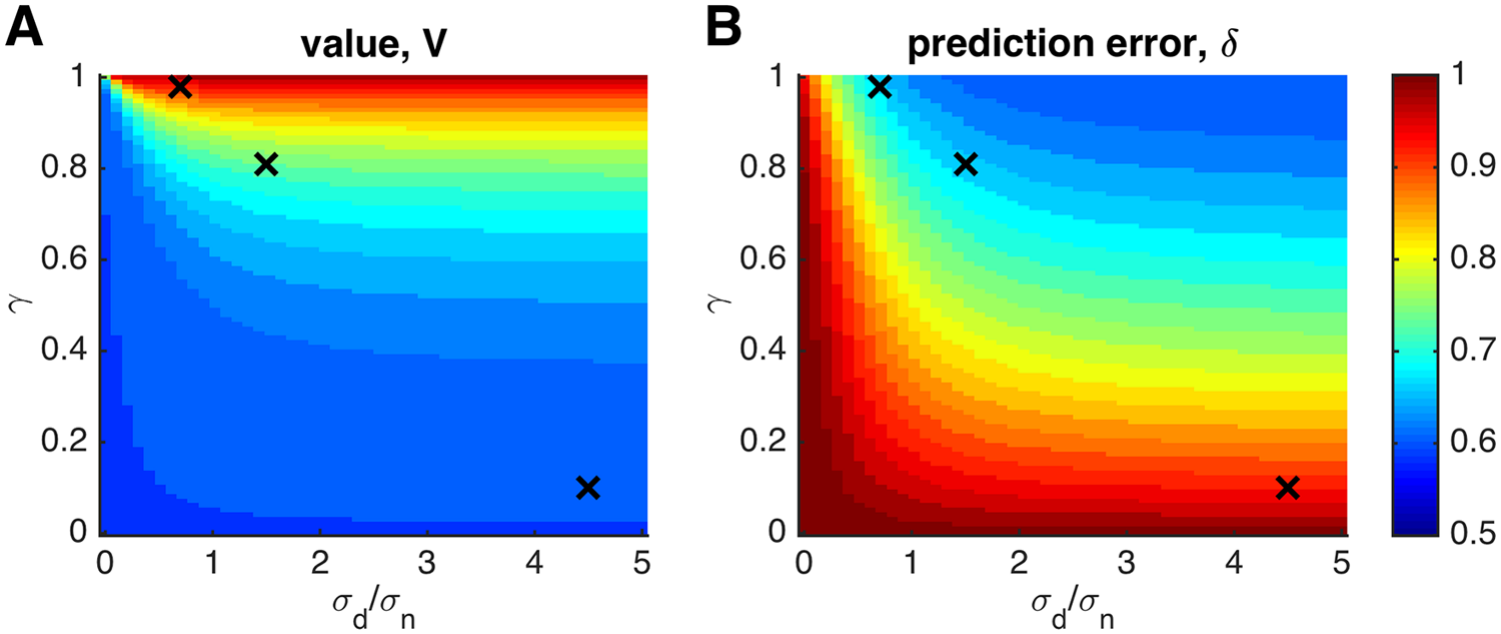
Insensitivity of value (A) and prediction error (B) regressors to the fit learning rate as a function of decay of the reward mean to zero, *γ*, and the drift variance to noise variance ratio of the reward mean, *σ*_*d*_/*σ*_*n*_, in experiments with drifting rewards. The three black crosses indicate the parameter values in the examples in Fig 7.

To explore the parameter space more thoroughly, we quantified the ‘insensitivity to learning rate’ as the fraction of (*α*_*g*_, *α*_*f*_)-space in which the correlations are greater than 0.7. This metric is 1 when the correlations are only weakly dependent on learning rate (as for the prediction error in the case of fixed rewards) and 0 when they are exquisitely sensitive. Fig 8 shows this metric as a function of the two parameters, *γ* and *σ*_*d*_/*σ*_*n*_, for the value and prediction error regressors. The plot demonstrates the somewhat reciprocal relationship between *ρ*(**V**_*g*_, **V**_*f*_) and *ρ*(**δ**_*g*_, **δ**_*f*_): when prediction errors have higher sensitivity to learning rate, the values tend to have lower sensitivity, and vice versa. Thus, while the sensitivity to learning rate can be tuned, there is a tradeoff between sensitivity to model-based regressors for prediction errors and value.

#### fMRI results

**Fig 9:**
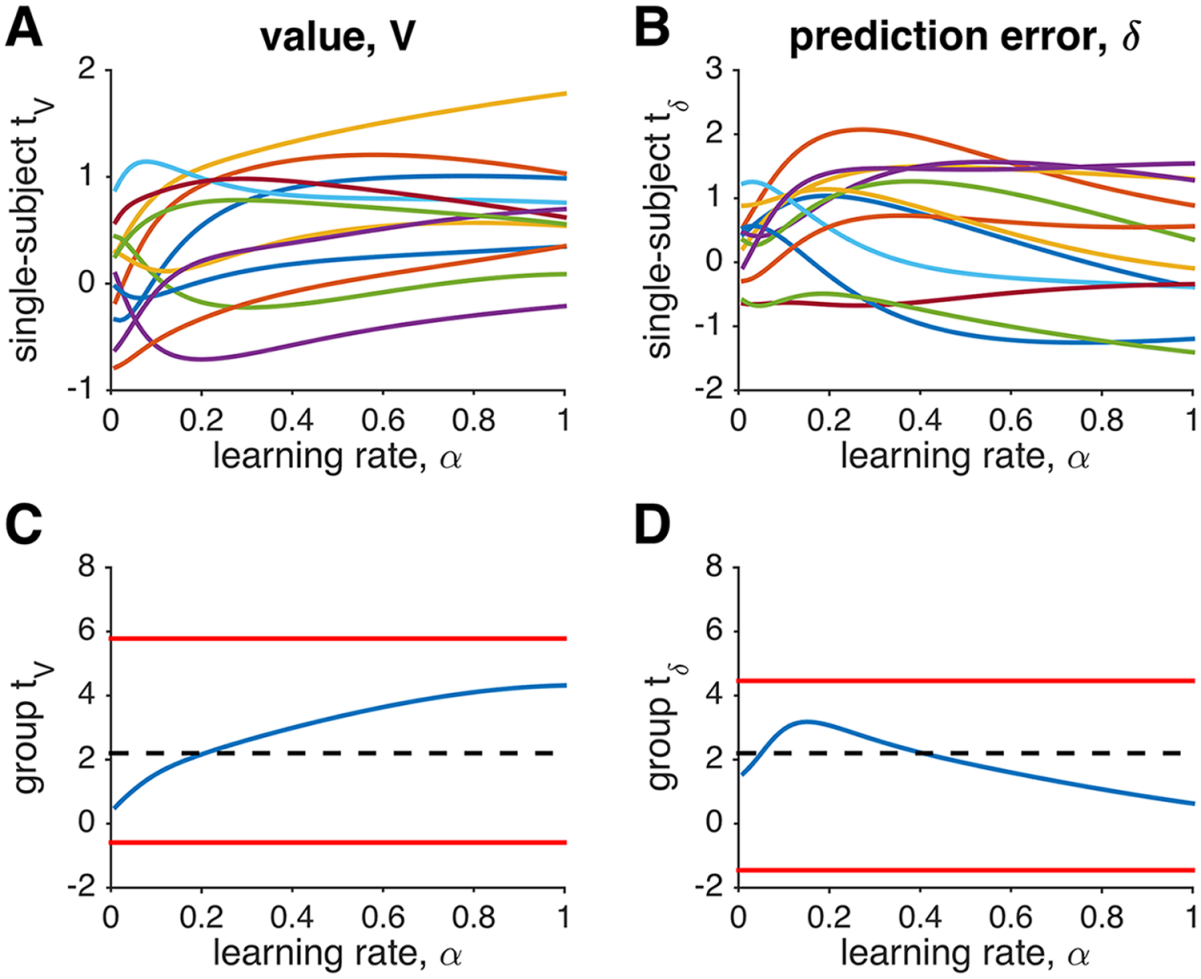
The effect of learning rate on the BOLD signal in vmPFC in [3], an experiment with drifting rewards. (A) Regression coefficients for value of the chosen option at the time of stimulus onset, as a function of learning rate. Each curve represents a single subject. (B) Single subject regression coefficients for prediction error at the time of reward. (C) Group analysis of value signals at the time of stimulus showing the group *t* statistic as a function of (group-wise) learning rate (blue). Red lines denote the best and worst case scenarios obtained by taking the value of the learning rate that either maximizes or minimizes the *t* statistic for each subject. Dashed black line: *p* = 0.05 threshold. (D) Group analysis of prediction error signals at the time of reward.

To test the theoretical predictions, we analyzed BOLD data from Daw et al. [3], performing a GLM analysis with a variety of different learning rates and examining value and prediction error regressors. The resulting regression coefficients and group *t* statistics are shown in Fig 9. Here we see much less sensitivity to the learning rate of the chosen value signal than the prediction error signal at both the single subject and group levels. This is in line with our predictions, as the reward parameters in the experiment (*γ* ≈ 0.98 and *σ*_*d*_/*σ*_*n*_ = 0.7) place it in the upper left of Fig 8, where values are more sensitive to the fit learning rate than are prediction errors.

A notable feature of these results is that the different regressors are significantly correlated with the neural signal in different regions of reward-parameter space, with prediction errors significantly correlated with BOLD signals when using low learning rates and values significant at higher learning rate. This reflects the fact that with low learning rates value changes slowly, and so prediction errors are more correlated with the (surprising and drifting) outcomes, whereas for high learning rates it is the value that closely tracks the drifting outcomes. It may not be surprising that the correlation between the different regressors and trial outcomes drives the significance of the regression result in vmPFC, as this area has been repeatedly associated with encoding of outcome magnitude [32, 33]. However, this result highlights again an important (and worrying) point: while the overall regression coefficients can be remarkably robust to the fit learning rate, interpretation of what function a neural area fulfills can change significantly as the fit parameter values change.

**Fig 10:**
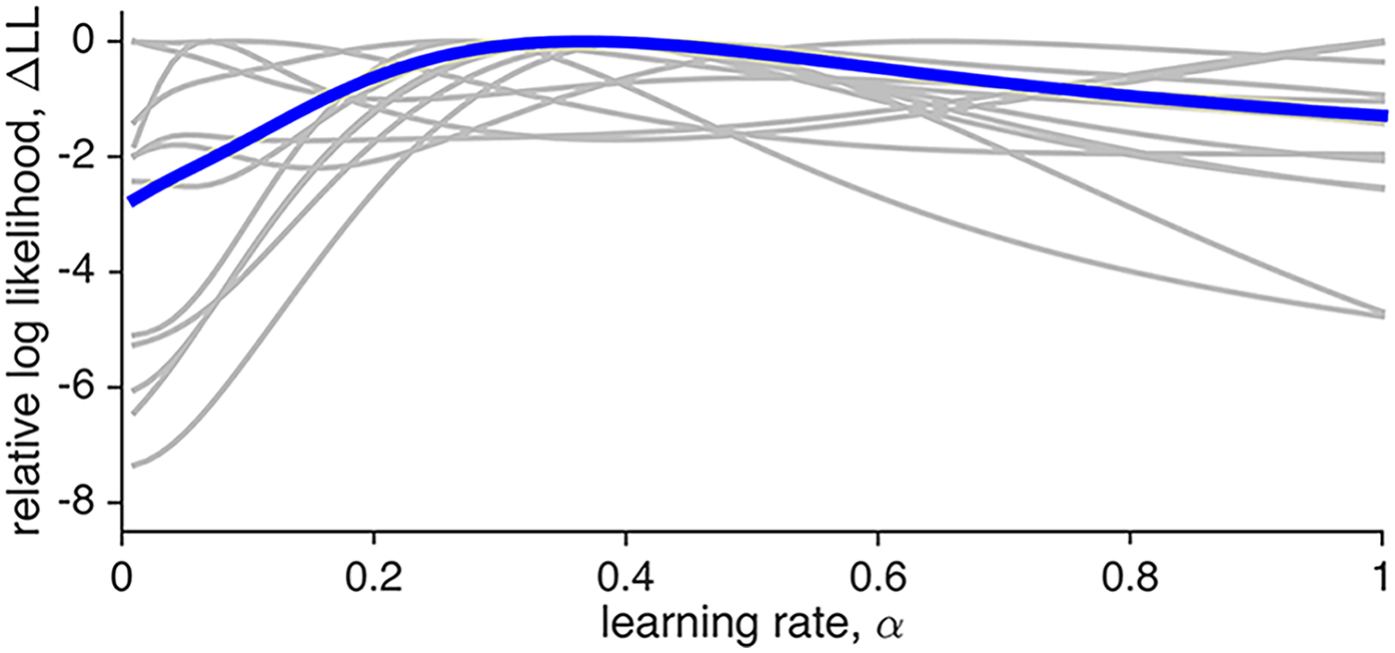
Fit of a linear model comprised of a single model-based regressor generated with different learning rates *α*, to fMRI data in the drifting reward experiment [3] as a function of learning rate. Each grey curve corresponds to a different subject and in blue is the mean across subjects. All curves are shifted to have a maximum value of 0. The y-axis is the same as that for Fig 5, to highlight the differences between the two experiments.

To further investigate the sensitivity of our results to settings of the learning rate, we again computed the log likelihood of the neural data for linear regression models using model-based regressors with different learning rates. These results are shown in Fig 10. Unlike the case of constant reward probability (Fig 5), in this experiment we found much stronger dependence of the log likelihood on learning rate, likely due to the increased contrast-to-noise ratio for the larger vmPFC ROI (11 here, compared to 0.4 for the NAc ROI). This increased sensitivity also allows us to extract a potentially meaningful fit of the learning rate to the fMRI data—on average 0.36 (± 0.08 [s.e.m.]). Of course, this analysis is only suggestive and one should be carefully interpreting group-averaged statistics.

To decide between different accounts of vmPFC activity—value, prediction error, or both—one could use a similar method to compare the goodness of fit of different models and assess, at the group level, which model fits the data best. In particular, one could compare three distinct linear models: one with a regressor for value but not prediction error, one with a regressor for prediction error but not value and one with both (for instance, generated using the best-fit learning rate). The log-likelihood measure (corrected for the different number of parameters, in this case, the number of regressors) could then be compared to determine the best model. We note that while such *model comparison* is closely related to the questions of parameter fitting and parameter estimation we consider here, it comes with none of the guarantees that we have established for parameter fitting.

## Discussion

In this paper, we considered the extent to which errors in the estimation of model parameters impact model-based fMRI. We showed that, in general, the answer to this question depends crucially on the correlation between regressors derived from different parameterizations of the model, *ρ*(**x**_*g*_, **x**_*f*_), and is further affected by the contrast-to-noise ratio in the data, CNR, and the number of trials, *T*, in the experiment. In the specific case where the fit parameter is the learning rate in a reinforcement learning model, we found that regressors for both value and prediction error signals were fairly insensitive to the fit learning rate, such that for realistic values of CNR and *T*, the results of the model-based analysis were predicted to be robust to different parameterizations. Indeed for an experiment with a fixed reward distribution, the estimated learning rate had close to no effect on the detection of prediction error signals in the NAc either in theory or in the experimental data. Similar results also held when rewards were drawn from a Gaussian distribution with a randomly drifting mean.

These findings are consistent with the report from one of the earliest model-based fMRI papers [18], in which changing the learning rate from 0.2 to 0.7 was found to have relatively little effect on the results. However, when either the contrast-to-noise ratio or number of trials is high, sensitivity of the model-based analysis to learning rate can increase. This might explain the anecdotal finding (personal communication, J.P. O’Doherty) that the results reported in Bray & O’Doherty [34] were relatively sensitive to learning rate. In particular, this study had more trials (*T* = 288) than in either [18] or [10] and also used ‘natural’ rewards (in the form of good- and bad-looking faces) instead of monetary rewards, which might lead to a larger effect and hence greater CNR.

Our results hold important consequences for the interpretation of model-based fMRI experiments. As regards learning rate, the relative insensitivity to this parameter is both good news and bad news. For studies investigating what areas in the brain are involved in reinforcement learning, these results are good news as the robustness to the fit parameters will make errors in the fitting procedure inconsequential. In this sense, our philosophy diverges slightly from that of Forstmann and colleagues [35] who suggest redesigning either the model or the experiment if parameters cannot be estimated with sufficient accuracy. In contrast, we espouse the position that imperfect parameter recovery can be tolerated if the scientific question of interest can be answered without it, as it can, for example, when we wish to know *where* reinforcement learning signals are located in the brain.

For studies that ask more nuanced questions, such as whether a particular signal is a reward signal, a value signal or a prediction error signal, or whether different areas use different learning rates, the insensitivity of the neural analysis to learning rate means that a simple analysis is not sufficient. In these cases, there is special premium for clever task design [29], and a more detailed analysis, for instance requiring that a putative neural prediction error signal correlate significantly with all its theoretical subcomponents [10]. Our analysis also suggests a way to minimize this problem: changing the experiment, either by optimizing the dynamics of the reward distribution or increasing the number of trials, can substantially change the sensitivity to learning rate.

The analysis we are suggesting bears resemblance to calculations of statistical power. Statistical power refers to the probability that a specific experiment will be successful in detecting an effect that truly exists—it is obvious why this is an important quantity to optimize in experiment design. Indeed many of the manipulations that we suggest—such as increasing the number of trials—will also improve statistical power. For cases in which the effect one is looking for involves differences in model parameters, we suggest a formula for testing in advance whether these differences are likely to be detectable neurally.

Of course, the fact that parameter values may be difficult to infer from brain data does not mean that they are not inferable at all. In many (if not all) cases, suitable behavioral data can provide strong constraints on model selection and parameter fitting. The ‘power’ of this type of analysis can also be tested, for example by recovering parameters from simulated data [36] and using data simulated by different models to test for confusion between these models [37]. Nevertheless, it is not obvious that parameters that provide a good description of behavior will necessarily correspond to processes in any brain area. For example, behavior could be driven by a combination of several distinct processes each with different parameter values [38–40].

More generally, for parameters other than the learning rate (for example, the discount factor in inter-temporal choice, or the softmax parameter in bandits tasks) our results highlight the importance of testing parameter sensitivity *before* running the experiment. This need not be done analytically (as was the case here) but can be approximated easily using simulations. As our results show, it is often possible to increase or decrease sensitivity to a particular variable by changing the parameters of the task and, with a clear focus on the goal of the model-based analysis, one could use such simulations to optimize experiment design.

Finally, while in this paper we have focused on the sensitivity of model-based fMRI to the parameters of a single model, an important question for future work is the extent to which fMRI can be used to adjudicate between *different* models. Such model comparison would involve computing goodness-of-fit measures (such as the log likelihoods we computed above) for each model and asking which model fit the fMRI data best. The extent to which models can be distinguished based on neural data is related to the degree of divergence of the predictions of the two models (i.e., the correlation between the regressors of the different models). However, it is also likely related to how close the compared models are to the ground-truth generative process that underlies the fMRI data, for which we unfortunately have no *a priori* guarantees.

## Supporting Information

S1 Text Detailed derivation of the statistics of the value and prediction error regressors. (PDF)

S2 Text Effect of number of trials, *T*, in real data. (PDF)

S1 Dataset NAc ROI data from [10]. (ZIP)
