# Supplementary material for: Is Model Fitting Necessary for Model-Based fMRI?
Source: PLoS Comput Biol. 2015 Jun 18;11(6):e1004237. doi: 10.1371/journal.pcbi.1004237 (PMC4472514; doi:10.1371/journal.pcbi.1004237)
Supplement: S1 Text — (PDF) [file pcbi.1004237.s001.pdf]

Supplementary Material
Is model fitting necessary for model-based fMRI?

Robert C. Wilson[1,*], Yael Niv[2]
[1] Department of Psychology and Cognitive Science Program, University of Arizona, Tucson AZ 85721
[2] Princeton Neuroscience Institute, Princeton University, Princeton NJ 08544
[*] E-mail: rcw2@princeton.edu

Detailed derivation of the statistics of the value and prediction error regressors

To derive the expressions for the correlations, $\rho(\mathbf{V}_i, \mathbf{V}_j)$ and $\rho(\boldsymbol{\delta}_i, \boldsymbol{\delta}_j)$, we need to compute the quantities: $\mu(\mathbf{V}_i)$, $\sigma(\mathbf{V}_i)$, $\text{cov}(\mathbf{V}_i, \mathbf{V}_j)$, $\mu(\boldsymbol{\delta}_i)$, $\sigma(\boldsymbol{\delta}_i)$, $\text{cov}(\boldsymbol{\delta}_i, \boldsymbol{\delta}_j)$. To do this we begin by noting that we can rewrite the values V_{it} as a weighted sum over all rewards (assuming $V_{i1} = 0$)

$$V_{it} = \sum_{a=1}^{t-1} \alpha_i (1-\alpha_i)^{t-a} r_a \tag{1}$$

This implies that the mean of the values is

$$\mu(\mathbf{V}_i) = \frac{1}{T} \sum_{t=1}^{T} \sum_{a=1}^{t-1} \alpha_i (1-\alpha_i)^{t-a} r_a \tag{2}$$

This can be rearranged into a more revealing form as follows

$$\begin{aligned}
\mu(\mathbf{V}_i) &= \frac{1}{T} \sum_{t=1}^{T} \left(\alpha_i r_{t-1} + \alpha_i (1-\alpha_i) r_{t-2} + \alpha_i (1-\alpha_i)^2 r_{t-3} + \ldots \right) \\
&= \frac{1}{T} \begin{pmatrix} \alpha_i r_{T-1} & + & \alpha_i (1-\alpha_i) r_{T-2} & + & \alpha_i (1-\alpha_i)^2 r_{T-3} & + & \ldots \\ +\alpha_i r_{T-2} & + & \alpha_i (1-\alpha_i) r_{T-3} & + & \alpha_i (1-\alpha_i)^2 r_{T-4} & + & \ldots \\ +\alpha_i r_{T-3} & + & \alpha_i (1-\alpha_i) r_{T-4} & + & \alpha_i (1-\alpha_i)^2 r_{T-5} & + & \ldots \\ +\ldots & & & & & & \end{pmatrix} \\
&= \frac{1}{T} \sum_{n=1}^{T} r_{T-n} \sum_{c=0}^{n} \alpha_i (1-\alpha_i)^c \\
&= \frac{1}{T} \sum_{n=1}^{T} r_{T-n} (1 - (1-\alpha_i)^{n+1}) \\
&= \frac{T-1}{T} \mu(\mathbf{r}) + \frac{1}{T} \sum_{n=0}^{T} r_{T-n} (1-\alpha_i)^{n+1} \\
&\approx \mu(\mathbf{r})
\end{aligned} \tag{3}$$

where the final approximation holds for large T. This shows that the mean of the values is approximately the mean of the rewards. From this it is straightforward to show that the mean of the prediction errors is zero at long T since,

$$\begin{aligned}
\mu(\boldsymbol{\delta}_i) &= \mu(\mathbf{r}) - \mu(\mathbf{V}_i) \\
&\approx \mu(\mathbf{r}) - \mu(\mathbf{r}) \\
&= 0
\end{aligned} \tag{4}$$

To compute the standard deviation and covariance of $\mathbf{V}_i$ we first compute $\mathbf{V}_i^T\mathbf{V}_j$,

$$
\begin{aligned}
\mathbf{V}_i^T\mathbf{V}_j &= \sum_{t=1}^{T}\sum_{a=1}^{t}\alpha_i(1-\alpha_i)^{t-a}r_a\sum_{b=1}^{t}\alpha_j(1-\alpha_j)^{t-b}r_b \\
&= \sum_{t=1}^{T}\alpha_i\alpha_j\begin{pmatrix} r_t^2 & +(1-\alpha_i)r_tr_{t-1} & +(1-\alpha_i)^2r_tr_{t-1} & +\ldots \\ +(1-\alpha_j)r_tr_{t-1} & +(1-\alpha_i)(1-\alpha_j)r_{t-1}^2 & +(1-\alpha_i)^2(1-\alpha_j)r_{t-1}r_{t-2} & +\ldots \\ +(1-\alpha_j)^2r_tr_{t-2} & +(1-\alpha_i)(1-\alpha_j)^2r_{t-1}r_{t-2} & +(1-\alpha_i)^2(1-\alpha_j)^2r_{t-2}^2 & +\ldots \end{pmatrix} \\
&= \sum_{t=1}^{T}\alpha_i\alpha_j\Bigg(\sum_{d=0}^{t-1}(1-\alpha_i)^d(1-\alpha_j)^d r_{t-d}^2 \\
&\qquad\qquad + \sum_{\Delta=0}^{t-1}\sum_{d=0}^{t-\Delta-1}(1-\alpha_i)^d(1-\alpha_j)^d\left((1-\alpha_i)^\Delta+(1-\alpha_j)^\Delta\right)r_{t-d}r_{t-d-\Delta}\Bigg) \\
&= \frac{\alpha_i\alpha_j}{T}\Bigg(\sum_{n=0}^{T-1}r_{T-n}^2\sum_{m=0}^{n}(1-\alpha_i)^m(1-\alpha_j)^m \\
&\qquad\qquad + \sum_{\Delta=1}^{T-1}\left((1-\alpha_i)^\Delta+(1-\alpha_j)^\Delta\right)\sum_{n=0}^{T-\Delta-1}r_{T-n}r_{T-n-\Delta}\sum_{m=0}^{n}(1-\alpha_i)^m(1-\alpha_j)^m\Bigg)
\end{aligned}
\tag{5}
$$

Now, this expression can be simplified by noting that

$$
\begin{aligned}
\sum_{n=0}^{T-1}r_{T-n}^2\sum_{m=0}^{n}(1-\alpha_i)^m(1-\alpha_j)^m &= \sum_{n=0}^{T-1}r_{T-n}^2\frac{1-(1-\alpha_i)^T(1-\alpha_j)^T}{1-(1-\alpha_i)(1-\alpha_j)} \\
&\approx \frac{\mu(\mathbf{r}^2)}{\alpha_i+\alpha_j-\alpha_i\alpha_j}
\end{aligned}
\tag{6}
$$

where the approximation holds for large T and $\mu(\mathbf{r}^2)$ is the mean square of the rewards. Likewise,

$$
\sum_{n=0}^{T-\Delta-1}r_{T-n}r_{T-n-\Delta}\sum_{m=0}^{n}(1-\alpha_i)^m(1-\alpha_j)^m \approx (T-\Delta)R_\Delta(\mathbf{r})
\tag{7}
$$

where $R_\Delta(\mathbf{r})$ is the autocorrelation of the reward signal at delay Δ,

$$
R_\Delta(\mathbf{r}) = \frac{1}{T-\Delta}\sum_{a=1}^{T-\Delta}r_ar_{a+\Delta}
\tag{8}
$$

Thus we can write

$$
\mathbf{V}_i^T\mathbf{V}_j \approx \frac{\alpha_i\alpha_j}{\alpha_i+\alpha_j-\alpha_i\alpha_j}\left(T\mu(\mathbf{r}^2)+\sum_{\Delta=1}^{T-1}\left((1-\alpha_i)^\Delta+(1-\alpha_j)^\Delta\right)(T-e)R_\Delta(\mathbf{r})\right)
\tag{9}
$$

This allows us to write the variance of $\mathbf{V}_i$ as

$$
\begin{aligned}
\sigma(\mathbf{V}_i)^2 &= \frac{\mathbf{V}_i^T\mathbf{V}_i}{T}-\mu(\mathbf{V}_i)^2 \\
&\approx \frac{\alpha_i}{2-\alpha_i}\left(\mu(\mathbf{r}^2)+2\sum_{\Delta=1}^{T-1}\left(1-\frac{\Delta}{T}\right)(1-\alpha_i)^\Delta R_\Delta(\mathbf{r})\right)-\mu(\mathbf{r})^2
\end{aligned}
\tag{10}
$$

and the covariance of $\mathbf{V}_i$ and $\mathbf{V}_j$ as

$$\begin{aligned} \mathrm{cov}(\mathbf{V}_i, \mathbf{V}_j) &= \frac{\mathbf{V}_i^T \mathbf{V}_j}{T} - \mu(\mathbf{V}_i)\mu(\mathbf{V}_j) \\ &\approx \frac{\alpha_i \alpha_j}{\alpha_i + \alpha_j - \alpha_i \alpha_j} \left(\mu(\mathbf{r}^2) + \sum_{\Delta=1}^{T-1} \left((1-\alpha_i)^{\Delta} + (1-\alpha_j)^{\Delta} \right) \left(1 - \frac{\Delta}{T} \right) R_\Delta(\mathbf{r}) \right) - \mu(\mathbf{r})^2 \end{aligned} \tag{11}$$

To compute $\boldsymbol{\delta}_i^T \boldsymbol{\delta}_j$ we note that, since $\boldsymbol{\delta}_i = \mathbf{r} - \mathbf{V}_i$, we can write

$$\boldsymbol{\delta}_i^T \boldsymbol{\delta}_j = \mathbf{r}^T \mathbf{r} - \mathbf{r}^T \mathbf{V}_i - \mathbf{r}^T \mathbf{V}_j + \mathbf{V}_i^T \mathbf{V}_j \tag{12}$$

Next we note that

$$\begin{aligned} \mathbf{r}^T \mathbf{V}_i &= \sum_{t=1}^{T} r_t \sum_{a=1}^{t} \alpha_i (1-\alpha_i)^{t-a} r_a \\ &= \sum_{\Delta=1}^{T-1} \alpha_i (1-\alpha_i)^{\Delta-1} \sum_{t=1}^{T-\Delta} r_t r_{t+\Delta} \\ &= \sum_{\Delta=1}^{T-1} \alpha_i (1-\alpha_i)^{\Delta-1} (T-\Delta) R_\Delta(\mathbf{r}) \end{aligned} \tag{13}$$

Thus, combining equations 9 and 13 we get

$$\begin{aligned} \boldsymbol{\delta}_i^T \boldsymbol{\delta}_j &= T\mu(\mathbf{r}^2) - \sum_{\Delta=1}^{T-1} (\alpha_i(1-\alpha_i)^{\Delta-1} + \alpha_j(1-\alpha_j)^{\Delta-1})(T-\Delta) R_\Delta(\mathbf{r})) \\ &\quad + \frac{\alpha_i \alpha_j}{\alpha_i + \alpha_j - \alpha_i \alpha_j} \left(T\mu(\mathbf{r}^2) + \sum_{\Delta=1}^{T-1} \left((1-\alpha_i)^{\Delta} + (1-\alpha_j)^{\Delta} \right) (T-\Delta) R_\Delta(\mathbf{r}) \right) \\ &= \frac{T(\alpha_i + \alpha_j)}{\alpha_i + \alpha_j - \alpha_i \alpha_j} \mu(\mathbf{r}^2) - \frac{(1-\alpha_i)(1-\alpha_j)}{\alpha_i + \alpha_j - \alpha_i \alpha_j} \sum_{\Delta=1}^{T-1} \left(\alpha_i^2 (1-\alpha_i)^{\Delta-1} + \alpha_j^2 (1-\alpha_j)^{\Delta-1} \right) (T-\Delta) R_\Delta(\mathbf{r}) \\ &= \frac{1}{\alpha_i + \alpha_j - \alpha_i \alpha_j} \left(T(\alpha_i + \alpha_j)\mu(\mathbf{r}^2) - \sum_{\Delta=1}^{T-1} \left(\alpha_i^2 (1-\alpha_i)^{\Delta-1} + \alpha_j^2 (1-\alpha_j)^{\Delta-1} \right) (T-\Delta) R_\Delta(\mathbf{r}) \right) \end{aligned} \tag{14}$$

This implies that the variance of the prediction errors is

$$\sigma(\boldsymbol{\delta}_i)^2 = \frac{2}{2-\alpha_i} \left(\mu(\mathbf{r}^2) - \sum_{\Delta=1}^{T-1} \alpha_i (1-\alpha_i)^{\Delta-1} \left(1 - \frac{\Delta}{T} \right) R_\Delta(\mathbf{r}) \right) \tag{15}$$

and the covariance is

$$\mathrm{cov}(\boldsymbol{\delta}_i, \boldsymbol{\delta}_j) = \frac{1}{\alpha_i + \alpha_j - \alpha_i \alpha_j} \left((\alpha_i + \alpha_j)\mu(\mathbf{r}^2) - \sum_{\Delta=1}^{T-1} \left(\alpha_i^2 (1-\alpha_i)^{\Delta-1} + \alpha_j^2 (1-\alpha_j)^{\Delta-1} \right) \left(1 - \frac{\Delta}{T} \right) R_\Delta(\mathbf{r}) \right) \tag{16}$$
