# Supplementary material for: Is Model Fitting Necessary for Model-Based fMRI?
Source: PLoS Comput Biol. 2015 Jun 18;11(6):e1004237. doi: 10.1371/journal.pcbi.1004237 (PMC4472514; doi:10.1371/journal.pcbi.1004237)
Supplement: S2 Text — (PDF) [file pcbi.1004237.s002.pdf]

Supplementary Material
Is model fitting necessary for model-based fMRI?

Robert C. Wilson[1,*], Yael Niv[2]
[1] Department of Psychology and Cognitive Science Program, University of Arizona, Tucson AZ 85721
[2] Princeton Neuroscience Institute, Princeton University, Princeton NJ 08544
[*] E-mail: bob@email.arizona.edu

Effect of number of trials, T, in real data

In the main paper, we provided a theoretical derivation of how the number of trials, T, should affect the single-subject t statistic. To show how this theoretical effect plays out in real data at the group level, we repeated the analyses used in Figures 4 and 9 in the main paper using only the first T trials from each experiment, for different values of T.

The results of this analysis are shown in figure S1 for the case of fixed reward probability [10], and in figure S2 for the case of a drifting reward [3]. In both cases, as expected, increasing the number of trials increases the group level t value and sharpens the dependence of the t statistic on the learning rate. This latter point is especially clear in figure S2, in which the original experiment involved more trials, and thus we were able to vary T over a wider range.

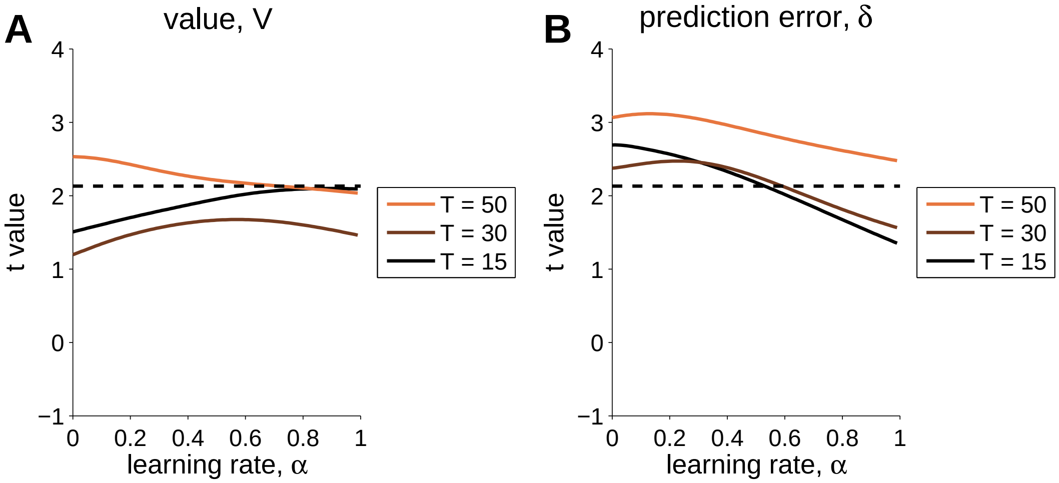

Figure S1. Effect of varying the number of trials, T, on the group level t statistic for data from [10]. Increasing T increases the group level t statistic for both value and prediction error.

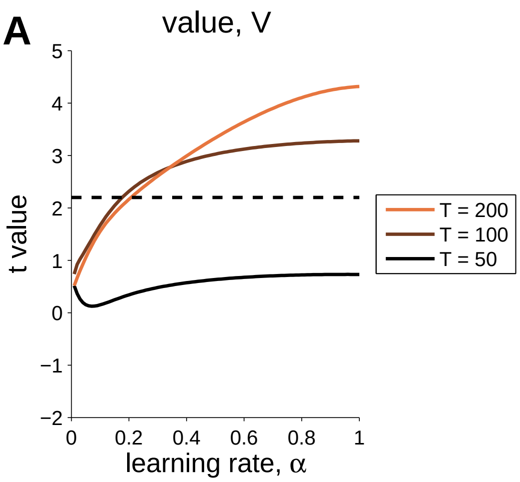

Figure S2. Effect of varying the number of trials, T, on the group level t statistic for data from [3]. Increasing T increases the group level t statistic for both value and prediction error. In addition, the t value becomes more strongly modulated by learning rate as T increases.

References

1. Niv Y, Edlund JA, Dayan P, O'Doherty JP (2012) Neural prediction errors reveal a risk-sensitive reinforcement-learning process in the human brain. The Journal of Neuroscience 32: 551–562.

2. Daw ND, O'Doherty JP, Dayan P, Seymour B, Dolan RJ (2006) Cortical substrates for exploratory decisions in humans. Nature 441: 876-879.
